# Prevalence and factors associated with hardcore smoking in Poland: Findings from the Global Adult Tobacco Survey (2009–2010)

**DOI:** 10.1186/1471-2458-14-583

**Published:** 2014-06-11

**Authors:** Dorota Kaleta, Bukola Usidame, Elżbieta Dziankowska-Zaborszczyk, Teresa Makowiec-Dąbrowska, Mall Leinsalu

**Affiliations:** 1 Department of Preventive Medicine, Medical University of Łódź, Łódź, Poland; 2 Department of Public Policy, McCormack Graduate School of Public Policy, University of Massachusetts, Boston, USA; 3 Department of Social and Preventive Medicine, Medical University of Łódź, Łódź, Poland; 4 Department of Work Physiology and Ergonomics, Nofer Institute of Occupational Medicine, Łódź, Poland; 5 Stockholm Centre on Health of Societies in Transition, Södertörn University, Huddinge, Sweden; 6 Department of Epidemiology and Biostatistics, National Institute for Health Development, Tallinn, Estonia

**Keywords:** Tobacco smoking, Hardcore smoker, Socio-demographic factors, Adults, GATS, Poland

## Abstract

**Background:**

Estimating the prevalence of hardcore smoking and identifying linked factors is fundamental to improve planning and implementation of effective tobacco control measures. Given the paucity of data on that topic, we aimed to assess the prevalence of and factors associated with hardcore smoking in Poland.

**Methods:**

We used data from the Global Adult Tobacco Survey (GATS). GATS is a representative, cross-sectional, household based survey conducted in Poland between 2009 and 2010. Binary logistic regression analysis was used to explore the associations of socio-demographic and smoking related variables with hardcore smoking among daily smokers.

**Results:**

The prevalence of hardcore smoking was 10.0% (13.0% among men and 7.3% among women) in the whole population of Poland at age 26 years and above. Hardcore smokers constitute 39.9% (41.6% among men and 37.7% among women) of all daily smokers in analyzed age frame. Being older, having started smoking at earlier ages, living in large cities (in women only), being less aware of negative health effects of smoking, having less restrictions on smoking at home was associated with higher risk of being hardcore smoker. Educational attainment and economic activity were not associated with hardcore smoking among daily smokers.

**Conclusions:**

High prevalence of hardcore smokers may be a grand challenge for curbing non-communicable diseases epidemic in Poland. Our findings should urge policy makers to consider hardcore smoking issues while planning and implementing tobacco control policies. Prevention of smoking uptake, education programs, and strengthening cessation services appeared to be the top priorities.

## Background

Tobacco use is one of the main risk factors for cancer, cardiovascular diseases and chronic lung diseases, also known as non-communicable diseases (NCDs) [1]. The risk of developing tobacco-related diseases increases proportionally with the consumption of tobacco products and the length of the smoking period [1]. Curbing the tobacco epidemic would be a huge step towards preventing the death and disability caused by cigarettes and other tobacco products. The status of the tobacco epidemic is a result of the number of people who start, stop and continue smoking. Early activities to combat tobacco smoking in Poland have been undertaken since the 1970s [2]. Over several decades the prevalence of smoking decreased substantially in Poland reaching to 33% in men and to 21% among women in 2010 [3]. Unfortunately, in the recent years the decreasing trend has stopped among the adult population [2]. Similar developments have been observed recently in many other developed countries. Despite of the multiple efforts to combat the tobacco epidemic, these efforts tend to hit a plateau after a significant reduction of smoking prevalence. One of the theories explaining this phenomenon is called “hardening hypothesis”. The “hardening hypothesis” suggests that as smoking prevalence decreases, lighter smokers will quit first, leaving more “hardcore” smokers in the population for whom smoking cessation may be especially difficult [4-6]. However, in some reports the hardening hypothesis is discounted, and there are suggestions that hardening occurs among treatment seekers, and there remains no clear evidence of hardening in the general population [4-8]. Some authors suggest that hardening occurs in the sense that, many of today's smokers possibly do have greater difficulty quitting, or are inherently less willing to do so when compared with earlier generations [9].

Given this, there is an urgent need to better understand the subpopulations of smokers, such as hardcore and non-hardcore smokers in order to improve and target tobacco control measures and to further reduce the smoking prevalence.

In line with this significant public health concern, the aim of this study was to assess the prevalence of hardcore smoking in Poland and to identify the factors associated with hardcore smoking in comparison with non-hardcore smoking.

## Methods

### Data

Data were derived from the Global Adult Tobacco Survey (GATS) [10]. The GATSs comprise a series of household based, cross-sectional, and nationally representative surveys based on global standard protocol for monitoring the use of tobacco among adult populations worldwide [11]. In Poland, the GATS was implemented in 2009–2010. The target population consisted of the non-institutional residents of Poland aged 15 years and older [10,12]. Selection process of the survey population was based on a multi-stage stratified geographically clustered sampling [13]. Out of the 14 000 households selected for the survey, 8948 (63.9%) households and 7840 (93.9%) sampled persons finalized the interviews. The overall survey participation rate was 65.1%. The questionnaires were completed during the face-to-face interviews at the respondents’ homes. The GATS data (also used in this study) are publicly available from the database of the Global Tobacco Surveillance System [http://www.cdc.gov/Tobacco/global/gtss/index.htm] and the methodology has been described in detail elsewhere [13]. After exclusion of respondents younger than 26 years, those of not being daily smokers and all cases with missing answers, the final sample used in this study consisted of 962 men and 665 women.

### Outcome variable

The measure of ‘hardcore’ smoking usually includes a mixture of motivational, dependence and behavioral variables [6,8,14]. Motivational and behavioral measures, such as intention to quit, may predict a failure of making quit attempts [15]. However, as some studies suggest, the dependence components best predict the continued smoking [15]. For the purpose of this study we differentiated hardcore and non-hardcore smokers among daily smokers (adult person who smokes regularly, at least one manufactured and/or hand-rolled cigarette a day). Hardcore smoker was defined as a current daily smoker who had been smoking for at least 5 years or longer, who smoked 15 cigarettes per day or more, had made no quit attempt in the past 12 months, and had no intention to stop smoking at all or in the next 12 months. Non-hardcore smoker was a current daily smoker who did not meet the other defining criteria of hardcore smoker.

### Smoking related characteristics

Hardcore and non-hardcore smokers were characterized by the age at smoking onset, awareness of the health consequences of smoking, nicotine dependence measures, rules regarding the smoking at home and the. Age at smoking onset reflected the age when smoking was started on a regular basis and was categorized into four groups: <14, 14-17, 18-20 and 21 years or older. Awareness of the health consequences of smoking was asked by the question: Do you think that tobacco smoking causes serious diseases? We classified respondents as being aware if they answered “yes”, and as unaware if they answered “no” or “do not know”. Three measures of nicotine dependence were covered: the mean number of cigarettes consumed per day, the time of the first cigarette after waking up (up to 30 minutes, 30 minutes and over) and waking up at night to smoke with answer categories “yes” and “no”. We also determined whether smokers had visited a healthcare professional (physician, nurse) in the year prior to the survey, and if they did, whether they had been asked about their smoking habits and whether they received quitting advice from the healthcare professional. Restrictions on smoking behavior at home were studied in four categories: smoking is allowed, no rules for smoking, smoking is prohibited with some exceptions, and smoking is completely prohibited. Finally, we evaluated the support for tobacco control policies among hardcore and non-hardcore smokers by differentiating between high, medium and low level support. This measure was based on eight questions specifying different items of tobacco control policies. The sum score was divided as supporting 6-8 policies (high level support), 3-5 policies (medium level) and 0-2 policies (low level).

### Socio-demographic variables

Age was analyzed in five groups: 26-29, 30-39, 40-49, 50-59, and ≥60 years old. For educational level respondents were classified as having completed the primary education, vocational education, secondary education, or higher education. Economic activity differentiated subjects who were currently employed, who were unemployed, and who were economically non-active (i.e. pupils, students, homemakers, retirees, and pensioners due to disability). We also determined respondents’ place of residence whether it was a rural or an urban area. For the latter we distinguished between urban areas up to 50 000, from 50 000 to 200 000, and over 200 000 inhabitants.

### Statistical analyses

All analyses were performed separately for men and women. Prevalence ratios with 95% confidence intervals were calculated for hardcore and non-hardcore smoking in general population at age 26 years and above. Hardcore smokers were compared to their non-hardcore counterparts with respect to the socio-demographic and smoking related characteristics. Chi-square test was used to assess the differences in the distribution of selected characteristics and t-test was performed to assess the differences in the mean values of continuous variables. To study the associations of selected socio-demographic and smoking related characteristics with hardcore smoking we used binary logistic regression analysis with results being presented as odds ratios (OR) with 95% confidence intervals. Because some of the independent variables were highly correlated we excluded these variables from the regression models [16]. Both univariable and multivariable analyses were performed. In multivariable analyses all selected socio-demographic and smoking related variables were simultaneously included to the model. Sample weights were used in all statistical analyses and the analyses were performed with Stata IC11 software.

## Results

Overall, there were 2,610 thousand hardcore smokers at age 26 years and above in Poland, i.e. about 10.0% of the whole population in this age frame (Table 1). Among men, the prevalence of hardcore smoking was 13.0% and among women the prevalence was 7.3%. Among daily smokers (data not shown), the prevalence of hardcore smoking was 41.6% for men (95% CI = 38.0–45.3) and 37.7% for women (95% CI = 33.4–41.9).

**Table 1:** Prevalence (PR) and 95% confidence intervals (CI) for hardcore and non-hardcore daily smoking at age 26 years and above in Poland, Global Adult Tobacco Survey in Poland (2009–2010)

	**Total**		**Men**		**Women**	
	**Non-hardcore PR (95% CI)**	**Hardcore PR (95% CI)**	**Non-hardcore PR (95% CI)**	**Hardcore PR (95% CI)**	**Non-hardcore PR (95% CI)**	**Hardcore PR (95% CI)**
Total	18.5 (17.4–19.6)	10.0 (9.2–10.8)	22.8 (21.1–24.5)	13.0 (11.7–14.4)	14.8 (13.4–16.1)	7.3 (6.3–8.3)
Age (years)						
26–29	21.8 (17.9–25.6)	7.3 (5.1–9.4)	26.0 (20.4–31.7)	8.7 (5.5–11.9)	17.5 (12.3–22.8)	5.8 (3.1–8.5)
30–39	18.9 (16.7–21.1)	11.5 (9.7–13.4)	22.1 (18.8–25.3)	16.7 (13.6–19.8)	15.7 (12.7–18.7)	6.3 (4.4–8.3)
40–49	23.7 (21.0–26.3)	14.1 (12.0–16.2)	27.5 (23.5–31.4)	15.6 (12.6–18.5)	19.8 (16.3–23.2)	12.5 (9.6–15.5)
50–59	23.7 (21.1–26.3)	13.7 (11.4–15.9)	26.0 (22.1–30.0)	15.8 (12.3–19.2)	21.6 (18.1–25.1)	11.8 (8.9–14.7)
≥60	9.3 (7.7–10.9)	3.9 (3.0–4.9)	14.6 (11.4–17.9)	6.4 (4.6–8.3)	5.8 (4.2–7.3)	2.3 (1.3–3.2)
Place of residence						
Rural	17.3 (15.9–18.8)	9.0 (7.9–10.2)	22.5 (20.2–24.8)	13.2 (11.4–15.1)	12.4 (10.6–14.2)	5.1 (3.9–6.3)
Urban	19.2 (17.8–20.8)	10.6 (9.4–11.7)	23.0 (20.6–25.4)	12.9 (11.0–14.7)	16.1 (14.3–18.0)	8.6 (7.2–10.0)

Prevalence and 95% confidence intervals were calculated using sample weights.

Table 2 compares characteristics for hardcore and non-hardcore smokers. Statistically significant differences in the distribution of hardcore and non-hardcore smokers were observed for most of the socio-demographic and smoking related characteristics except for education, economic activity, seeing health provider (women only), health provider asking if smokes and health provider giving advices. Hardcore smokers were more likely to be in the 30–59 age group among men and in the 40–59 age group among women, more likely to have started regular smoking before age 18, to have primary or secondary education (in women), be economically active, live in medium-size (men only) or large (women) cities, and to be less aware of health consequences of smoking. Hardcore smokers were more nicotine dependent: they were more likely to have their first cigarette up to 30 minutes after waking and to wake up at night to smoke. They were also less likely to have seen a health provider over the past year and were less likely been asked about their smoking. Compared to non-hardcore smokers hardcore smokers lived more often at homes were smoking was allowed or were no rules were established and they were less supportive for tobacco control policies. Among men the mean age at smoking onset was one year younger among hardcore smokers compared to non-hardcore smokers (17.9 vs. 18.9 years respectively), among women the difference was approximately one and a half year (19.3 vs. 20.9). The mean number of cigarettes smoked per day for male hardcore smokers was 22.3 compared to 16.4 among non-hardcore smokers; among women the respective numbers were 20.8 and 12.8.

**Table 2:** Characteristics for hardcore and non-hardcore daily smokers at age 26 years and above, Global Adult Tobacco Survey in Poland (2009–2010)

	**Men**			**Women**		
	**Non-hardcore smokers, %**	**Hardcore smokers, %**	**p-value for differences**^**a**^	**Non-hardcore smokers, %**	**Hardcore smokers, %**	**p-value for differences**^**a**^
Age (years)			<0.001			<0.05
26–29	13.7	7.3		12.3	7.5	
30–39	21.4	28.3		21.6	17.2	
40–49	27.1	27.1		24.2	33.8	
50–59	22.9	25.6		29.5	33.3	
≥ 60	15.0	11.7		12.4	8.2	
Age at smoking onset			<0.001			<0.001
< 14	3.1	4.0		0.7	0.7	
14–17	32.1	42.3		21.6	33.5	
18–20	43.0	39.8		42.6	42.4	
≥ 21	21.7	13.9		35.0	23.4	
Education			NS			NS
Primary	14.2	14.2		11.3	14.1	
Vocational	45.8	44.7		31.1	28.6	
Secondary	32.3	33.5		41.2	46.9	
High	7.8	7.6		16.3	10.5	
Economic activity			NS			NS
Employed	63.9	66.2		54.7	61.1	
Unemployed	13.2	13.0		5.8	4.8	
Not active	22.9	20.8		39.5	34.2	
Place of residence			<0.01			<0.01
Rural	39.9	40.3		29.4	24.3	
Urban up to 50 000	27.7	21.4		24.1	21.1	
50 000–200 000	12.4	19.7		21.8	16.7	
Over 200 000	20.0	18.6		24.7	37.9	
Awareness of health consequences of smoking			<0.001			<0.001
Yes	85.6	74.5		88.1	76.0	
No	14.4	25.5		11.9	24.0	
Time for first cigarette			<0.001			<0.001
Up to 30 min	57.9	79.4		46.5	78.1	
30 min or later	42.1	20.6		53.5	21.9	
Wake up at night to smoke			<0.001			<0.001
Yes	20.3	36.6		15.7	37.2	
No	79.7	63.4		84.3	62.8	
Saw health provider in last year			<0.001			NS
Yes	59.5	47.1		72.6	65.8	
No	40.5	52.9		27.4	34.2	
Health provider asked if smokes tobacco^b^			NS			NS
Yes	63.9	58.3		64.3	56.8	
No	36.1	41.7		35.7	43.2	
Health provider advised to quit^c^			NS			NS
Yes	75.3	79.0		78.7	77.1	
No	24.7	21.0		21.3	22.9	
Rules about smoking at home			<0.001			<0.001
Smoking allowed	41.4	58.7		45.2	61.6	
No rules	8.2	8.8		11.0	12.7	
Smoking prohibited with some exceptions	27.9	22.0		31.8	21.5	
Smoking completely prohibited	22.4	10.4		12.1	4.2	
Support for tobacco control policies			<0.001			<0.001
High	50.9	37.2		49.8	28.6	
Medium	31.0	44.8		40.1	49.6	
Low	18.1	18.0		10.2	21.8	
Mean age of smoking onset in years (SE)	18.9 (0.185)	17.9 (0.192)	<0.001	20.9 (0.308)	19.3 (0.256)	<0.001
Mean number of cigarettes per day (SE)	16.4 (0.453)	22.3 (0.418)	<0.001	12.8 (0.334)	20.8 (0.380)	<0.001
	*n* = 539	*n* = 423		*n* = 426	*n* = 239	

Percentages and mean values were calculated using sample weights.

^a^P-values for differences between hardcore and non-hardcore smokers. Chi-square test is used for categorical variables, t-test for continuous variables; NS = not significant.

^b^Restricted to respondents who saw health provider within past year.

^c^Restricted to respondents who saw health provider within past year and were asked whether they smoke.

SE = standard error.

Results from the regression analyses among daily smokers are presented in Table 3 (for men) and Table 4 (for women). Univariable analyses showed that men in the 30–59 age group and women in the 40–49 age group had statistically significantly higher odds of being hardcore smokers compared to the youngest age group. Statistically significant association with hardcore smoking was also found for men and women who started smoking at age 14-17 compared to those who started smoking after age 20. Compared to the rural residents, men living in the medium-size cities and women living in the cities with over 200 000 inhabitants had higher odds of being hardcore smokers. Being unaware of the health consequences of smoking was related to more than two-fold risk of being hardcore smoker in both men and women. A clear gradual association was found for rules regulating smoking at home with those daily smokers who lived at homes where smoking was allowed having three- (in men) and nearly four-fold (in women) risk of being hardcore smoker compared to those who lived at homes were smoking was completely prohibited. No statistically significant associations were found for education and economic activity.

**Table 3:** Odds ratios (OR) with 95% confidence intervals (CI) for hardcore smoking by selected characteristics in men at age 26 years and above, Global Adult Tobacco Survey in Poland (2009–2010)

	**Univariable model OR (95% CI)**	**Multivariable model**^**a **^**OR (95% CI)**
Age (years)		
26–29	1.00	1.00
30–39	2.47 (1.43–4.29)	2.99 (1.68–5.32)
40–49	1.87 (1.08–3.24)	2.32 (1.28–4.19)
50–59	2.08 (1.19–3.67)	2.26 (1.22–4.16)
≥ 60	1.45 (0.77–2.73)	1.61 (0.72–3.58)
Age at smoking onset		
< 14	1.99 (0.72–5.48)	2.96 (1.08–8.12)
14–17	2.07 (1.32–3.24)	2.25 (1.41–3.58)
18–20	1.45 (0.93–2.26)	1.57 (1.01–2.45)
≥ 21	1.00	1.00
Education		
Primary	1.02 (0.52–1.97)	0.77 (0.36–1.64)
Vocational	0.99 (0.54–1.80)	0.74 (0.39–1.41)
Secondary	1.05 (0.57–1.96)	0.92 (0.48–1.75)
High	1.00	1.00
Economic activity		
Employed	1.00	1.00
Unemployed	0.95 (0.58–1.55)	0.99 (0.59–1.66)
Not active	0.88 (0.61–1.26)	0.96 (0.57–1.64)
Place of residence		
Rural	1.00	1.00
Urban up to 50 000	0.76 (0.51–1.14)	0.70 (0.46–1.07)
50 000–200 000	1.57 (1.02–2.41)	1.27 (0.79–2.04)
Over 200 000	0.92 (0.60–1.40)	0.79 (0.51–1.24)
Awareness of health consequences of smoking		
Yes	1.00	1.00
No	2.03 (1.39–2.98)	1.81 (1.20–2.73)
Rules regarding smoking at home		
Smoking allowed	3.05 (1.92–4.84)	2.97 (1.85–4.75)
No rules	2.31 (1.23–4.32)	2.26 (1.19–4.29)
Smoking prohibited with some exceptions	1.70 (1.02–2.84)	1.67 (0.99–2.81)
Smoking completely prohibited	1.00	1.00

Odds ratios were calculated using sample weights.

^a^Mutually adjusted for all variables.

**Table 4:** Odds ratios (OR) with 95% confidence intervals (CI) for hardcore smoking by selected characteristics in women at age 26 years and above, Global Adult Tobacco Survey in Poland (2009–2010)

	**Univariable model OR (95% CI)**	**Multivariable model**^**a **^**OR (95% CIn**
Age (years)		
26–29	1.00	1.00
30–39	1.31 (0.64–2.70)	1.62 (0.78–3.35)
40–49	2.30 (1.16–4.56)	2.73 (1.34–5.56)
50–59	1.86 (0.93–3.70)	2.29 (1.08–4.86)
≥ 60	1.09 (0.49–2.41)	1.53 (0.63–3.70)
Age at smoking onset		
< 14	1.56 (0.14–17.75)	4.05 (0.49–33.08)
14–17	2.31 (1.41–3.80)	2.62 (1.53–4.48)
18–20	1.49 (0.96–2.33)	1.84 (1.13–3.01)
≥ 21	1.00	1.00
Education		
Primary	1.94 (0.95–3.96)	2.11 (0.94–4.73)
Vocational	1.43 (0.76–2.70)	1.37 (0.71–2.66)
Secondary	1.77 (0.98–3.22)	1.74 (0.94–3.23)
High	1.00	1.00
Economic activity		
Employed	1.00	1.00
Unemployed	0.74 (0.35–1.56)	0.73 (0.32–1.69)
Not active	0.77 (0.53–1.14)	0.80 (0.49–1.31)
Place of residence		
Rural	1.00	1.00
Urban up to 50 000	1.06 (0.63–1.79)	0.91 (0.51–1.62)
50 000–200 000	0.93 (0.55–1.57)	0.77 (0.43–1.38)
over 200 000	1.86 (1.18–2.92)	1.80 (1.10–2.96)
Awareness of health consequences of smoking		
Yes	1.00	1.00
No	2.35 (1.44–3.85)	2.22 (1.29–3.83)
Rules regarding smoking at home		
Smoking allowed	3.90 (1.54–9.85)	3.57 (1.33–9.55)
No rules	3.32 (1.15–9.57)	1.89 (0.61–5.84)
Smoking prohibited with some exceptions	1.93 (0.73–5.09)	1.74 (0.62–4.87)
Smoking completely prohibited	1.00	1.00

Odds ratios were calculated using sample weights.

^a^Mutually adjusted for all variables.

In multivariable analyses, the effect of age and age at smoking onset on hardcore smoking became even stronger. Men in the 30–59 age group and women in the 40-49 age group had more than two times higher odds compared to the youngest age group; with highest odds found for men in the 30-39 age group (OR = 2.99) and for women in the 40-49 age group (OR = 2.73). The association with age at smoking onset became clearly gradual, younger age at smoking onset was related to higher odds of becoming hardcore smoker. Starting smoking before age 14 was associated with nearly three- and four-fold risk respectively in men and women (although statistically not significant in women). For place of residence, awareness of health consequences and rules regarding smoking at home all associations with hardcore smoking were slightly attenuated in multivariable analyses (to the statistically insignificant level for men according to the place of residence).

## Discussion

In the studied period, 10.0% of the population of Poland, and 41.6% of male and 37.7% of female daily smokers at age 26 years or older were hardcore smokers. The prevalence of hardcore smokers in the general population and among daily smokers found in GATS Poland seems to be the highest that has been described in the literature until now [15,17]. Kishore et al., analyzing data from GATS conducted in three South-East Asian countries revealed that hardcore smokers constitute between 3.1% in India to 6.0% in Thailand of the adult population which translates into 18.3% in India and into 29.7% in Thailand of daily smokers [17]. Ferketich et al. also estimated that 7.8% of all adult Italians and respectively 33.1% of all smokers in Italy in 2007 were hardcore smokers [15]. Lower prevalence of hardcore smoking among daily smokers was also registered in the US (California (5.2%) and Missouri (7.8%)), England (16%), and Norway (25.0%) [17-20]. The factors that may lie behind these huge disparities in hardcore smoking between countries are difficult to explain except that different studies use different study designs, or focus on selected groups like urban population, patients etc. The different definitions of hardcore smokers may also limit the ability to compare results across studies [14]. Aside methodological issues, countries also differ significantly in terms of their economic, social and cultural context which may play vital roles in influencing the tobacco epidemic phase. Also, the tobacco control environment, access to education or cessation services can be considered in this context.

In GATS Poland, the odds of being a hardcore smoker varied across age groups as in many other studies [16,17,19]. However in many studies, a higher concentration of hardcore smokers was observed in older age groups like 65 years or older [16,19,21]. Our results showed that the highest odds for hardcore smoking were found among subjects younger than 65 years, in men in the 30–59 age group and in women in the 40–59 age group. We can speculate that at a younger age men and women are less likely to consider smoking cessation than at age 60 and above, due to relatively good health and an absence of alarming symptoms caused by intensive tobacco use.

Consistent with other studies, GATS displayed that young age at smoking onset is strongly related with hardcore smoking [15,16,18,22] The hardcore smokers became regular smokers at a younger age than non-hardcore smokers [16]. Smoking causes nicotine addiction over time [23]. The earlier one starts smoking, the greater the risk of dependence, heavy smoking or difficulty with quitting as an adult [16,22,24]. Currently, Polish hardcore smokers appear to be mainly a cohort of middle-aged men and women. However, the recent studies have shown that adolescents are experimenting with cigarettes at relatively younger ages which may indicate that a new, younger cohort of highly addicted individuals may be budding [2,16]. Declining age of smoking initiation is alarming because negative health impact of smoking will be probably larger in the young cohorts of today [25,26]. Nonetheless, Emery et al. concluded that even if adolescent smoking increases beyond current levels, it is likely that smoking rates will continue to decline over the next 2 to 3 decades as the current cohort of older smokers diminishes through death and quitting [16]. Programs that delay smoking initiation might have considerable value even if they do not succeed in fully preventing the uptake of smoking [24]. Delaying smoking initiation among adolescents could eventually reduce the rate of heavy or hardcore smoking and increase the potential for successful cessation [24].

GATS Poland revealed that participants who were unaware of the health risks of smoking were more likely to be hardcore smokers compared to those aware. Most importantly, some reports showed that the knowledge on the harmful risks of tobacco use is still insufficient in Poland [27]. Health knowledge thus seems to be one of the most important factors that might prevent hardcore smoking. There is a need for better education on the risks of smoking which includes, improving the overall knowledge of quitting benefits and reduction of health risks related to smoking cessation [3]. This knowledge is often delivered through the contacts with health care professionals [28]. Brief interventions – doctors advising patients to quit has been considered the simplest approach to increase smoking cessation [28]. Our results (although in most cases on statistically insignificant level) showed that hardcore smokers tended to have less visits to health care professionals and those who visited were less likely of being asked about their smoking habits or advised to quit when compared to non-hardcore smokers.

The main focus of smoking bans in public places or indoor areas is to protect nonsmokers from exposure to environmental tobacco smoke. The added value of this strategy is that it often increases the likelihood of quitting among smokers and is considered as a key factor associated with cessation attempts and success [18,29-34]. There is a strong and consistent population-level evidence that smoke-free homes are associated with increased smoking cessation and decreased cigarette consumption in adult smokers [35,36]. As Mills et al. reported, both longitudinal and cross-sectional studies have revealed that smokers who had or who newly implemented a smoke-free home were significantly more likely to make a quit attempt and to be abstinent, after controlling for confounding factors. In longitudinal studies, those who continued to smoke had a modest, but significant, decrease in cigarette consumption at follow-up [36]. Findings from GATS Poland indicated that hardcore smokers were less likely to have smoke-free rules in their homes relative to non-hardcore smokers. Underestimation of the need to protect non-smokers at home or elsewhere from tobacco smoke is also supported by our finding that hardcore smokers were generally less supportive for tobacco control policies. Intensive and comprehensive tobacco control campaigns addressing smoke-free policies are thus urgently needed in Poland in order to curb the tobacco epidemic.

### Study limitations

For the purpose of this study we selected subjects who were 26 years or older at the time of the survey. Individuals aged 25 years and younger were excluded from the analysis because they still might have been engaged in the process of smoking uptake and therefore may not have reached a stable level of average daily consumption or solidified their intentions regarding quitting [16]. The GATS was carefully designed, nonetheless contains some limitations [10]. Some potential limitations are associated with the use of self-reports and a cross-sectional design, that have been broadly discussed in previous papers and should not significantly decrease the value of the study [3,12]. Nevertheless, we admit that because of the cross-sectional design, the direction of any causal association cannot be established in this study. It should also be highlighted that we examined the prevalence of and factors associated with hardcore smoking in Poland using component construct information available from GATS standard questionnaire, and we therefore may have missed some important characteristics (e.g. household income, mental health problems and alcohol or other addictive substance use) that could have impact on hardcore smoking. This missing information does not allow comparing GATS data with many other studies and thus requires a serious consideration in designing the future surveys.

## Conclusions

Hardcore smokers constitute a large population of established smokers in Poland which is challenging from the public health perspective. Although the hardcore smokers are at an increased risk of tobacco-related diseases, they are resistant to quit and respond less to tobacco control activities [7,16]. Without intensive interventions targeted to hardcore smokers, we may face difficulties in substantially increasing cessation rates and achieving sufficient progress in control of tobacco related diseases in Poland.

These findings should urge policy makers and relevant stakeholders to consider hardcore smoking issues while planning and implementing tobacco control policy in Poland. Prevention of smoking uptake, health education such as information programs on health consequences of smoking together with informing people about benefits of quitting, and strengthening cessation service are the top priority.

## Competing interests

The authors declare no conflicts of interest.

## Authors’ contributions

DK as a representative of the World Health Organization (WHO CO POL) coordinated GATS in Poland, outlined the paper, discussed core ideas, and prepared the final manuscript. BU did literature search. EDZ prepared the dataset, did the initial data analysis. TMD commented on drafts. ML conducted the final statistical analysis and contributed intellectually to the final manuscript. All authors read and approved the final manuscript.

## Pre-publication history

The pre-publication history for this paper can be accessed here:

http://www.biomedcentral.com/1471-2458/14/583/prepub
